# The Density and Length of Filopodia Associate with the Activity of Hyaluronan Synthesis in Tumor Cells

**DOI:** 10.3390/cancers12071908

**Published:** 2020-07-15

**Authors:** Heikki Kyykallio, Sanna Oikari, María Bueno Álvez, Carlos José Gallardo Dodd, Janne Capra, Kirsi Rilla

**Affiliations:** Institute of Biomedicine, School of Medicine University of Eastern Finland, Yliopistonranta 1E, 70211 Kuopio, Finland; heikki.kyykallio@uef.fi (H.K.); sanna.oikari@uef.fi (S.O.); buenoalvezm@gmail.com (M.B.Á.); cgallardo.dodd@gmail.com (C.J.G.D.); janne.capra@uef.fi (J.C.)

**Keywords:** hyaluronan, filopodia, CD44, hyaluronan synthase, cancer

## Abstract

Filopodia are multifunctional finger-like plasma membrane protrusions with bundles of actin filaments that exist in virtually all cell types. It has been known for some time that hyaluronan synthesis activity induces filopodial growth. However, because of technical challenges in the studies of these slender and fragile structures, no quantitative analyses have been performed so far to indicate their association with hyaluronan synthesis. In this work we comprehensively address the direct quantification of filopodial traits, covering for the first time length and density measurements in a series of human cancer cell lines with variable levels of hyaluronan synthesis. The synthesis and plasma membrane binding of hyaluronan were manipulated with hyaluronan synthase 3 (HAS3) and hyaluronan receptor CD44 overexpression, and treatments with mannose, 4-methylumbelliferone (4-MU), and glucosamine. The results of this work show that the growth of filopodia was associated with the levels of hyaluronan synthesis but was not dependent on CD44 expression. The results confirm the hypothesis that abundance and length of filopodia in cancer cells is associated with the activity of hyaluronan synthesis.

## 1. Introduction

Different plasma membrane-covered cellular protrusions supported by the actin cytoskeleton are displayed by all cell types, reflecting and regulating their biological functions [1]. Filopodia are thin, finger-like protrusions (Ø 0.1–0.3 μm) involved in a great range of cellular processes including cell migration, wound healing, chemoattractant guidance [2], extracellular matrix adhesion and remodeling [3], and embryonic development [4]. Filopodia and similar protrusions have important cellular functions in several different cell types. Intestinal epithelial cell microvilli closely resemble filopodia, and in addition to actin, other common proteins are present in both structures [5]. In neurons, filopodia act as precursors to dendritic branches [6]. In cancer cells, the number of filopodia is often increased, which enhances the migration and invasiveness of cancer cells. Filopodia have been shown to increase metastasis of several different cancer cell types [7]. Actin bundling proteins present in filopodia are generally overexpressed in cancers, which increases the length and number of filopodia. This results in the increased ability of cells to migrate and invade, which generally correlates with poor survival prognosis [8,9].

Hyaluronan (HA) is one of the main components of the extracellular matrix of normal and diseased tissues. It is synthesized by hyaluronan synthases (HAS) located in the plasma membrane, and bound by specific receptors, such as transmembrane adhesion protein CD44 [10]. Our research group has shown that hyaluronan synthesis activity induces growth of extremely long plasma membrane protrusions [11,12] that resemble filopodia [13]. Recent studies also suggest that hyaluronan synthesis activity of cells is correlated with abundance and length of filopodia [14]. Furthermore, there is strong evidence that hyaluronan-positive filopodia act as specific hyaluronan factories that are mechanically supported by hyaluronan [13,15,16]. However, the analyses of filopodia are limited because of their slenderness and small size, lability, and fragility. Due to these challenges, no quantitative studies on the role of hyaluronan synthesis activity or hyaluronan receptors on filopodial growth are currently available.

Until not long ago, eye-based filopodia detection followed by laborious manual tracing with appropriate manipulation software was considered the only way to quantitatively analyze filopodia from confocal microscopy images [17]. However, several computer-based automatic or semi-automatic approaches have been developed in the recent years for filopodia detection. Relevant platforms FiloDetect [17] and CellGeo [18] remain limited by proprietary software MATLAB requirements, while others such as ADAPT [19] are freely available ImageJ supported plugins with relatively similar properties. ImageJ plugin FiloQuant [20] is one of the latest automated quantification tools developed for filopodia, suitable for cancer cell cultures.

The aim of this study is to investigate whether the hyaluronan secretion levels correlate with filopodial growth, by using three different cancer cell lines and manipulation of their hyaluronan metabolism by stable or transient transfections of hyaluronan synthase 3 (HAS3) and CD44 and regulating the supply of sugar precursors. In this work we show for the first time comprehensive and quantitative data on hyaluronan-dependent filopodia. The results of this study indicate that filopodia formation correlates with hyaluronan synthesis activity but is not dependent on CD44 expression.

## 2. Materials and Methods

### 2.1. Cell Culture

The MCF-7 human breast adenocarcinoma cell line with inducible GFP-HAS3 expression [21] was cultured in minimum essential medium alpha (MEMα, EuroClone, Pavia, Italy) supplemented with 5% FBS (Gibco heat-inactivated fetal bovine serum, Thermo Fisher Scientific, Waltham, MA, USA), 2 mM glutamine (EuroClone), 100 µg/mL streptomycin sulfate, and 100 U/mL penicillin (EuroClone). For maintenance, the culture medium was supplemented with 50 µg/mL hygromycin B, but during the experiments, cells were grown without hygromycin B. To induce GFP-HAS3 overexpression, 0.5 μg/mL of doxycycline (doxycycline hydrochloride, Sigma, St. Louis, MO, USA) was used. Cells were passaged twice a week at a 1:5 split ratio using 0.05% trypsin (w/v) 0.02% EDTA (w/v) (Biochrom AG, Berlin, Germany).

MKN74 human gastric cancer cell lines, MOCK and CD44 [22] were cultured in RPMI 1640 medium (Lonza, Walkersville, MD, USA) supplemented with 10% FBS (Thermo Fisher Scientific), 100 μg/mL streptomycin sulphate (EuroClone), and 100 U/mL penicillin (EuroClone). Both cell lines were passaged twice a week at a 1:5 split ratio using 0.05% trypsin (w/v) 0.02% EDTA (w/v) (Biochrom AG, Berlin, Germany).

MCF10 human breast cell lines, MCF10A and MCF10CA, were cultured in DMEM/F12 medium (Gibco) supplemented with 5 % horse serum (Invitrogen, Carlsbad, CA, USA), 2 mM glutamine (EuroClone), 100 µg/mL streptomycin sulfate, 100 U/mL penicillin (EuroClone), 0.5 µg/mL epidermal growth factor (EGF, Sigma), 0.5 µg/mL hydrocortisone (Sigma), 0.1 µg /mL cholera toxin (Sigma), and 10 µg/mL insulin (Sigma). Both cell lines were passaged twice a week at the following split ratios (MCF10CA 1:20; MCF10A 1:25) using 0.05% trypsin (w/v) 0.02% EDTA (w/v) (Biochrom AG, Berlin, Germany).

For treatments of cells, mannose was used at 20 mM final concentration, 4-methylumbelliferone (4-MU) at 0.5 mM, and glucosamine at 0.5–2.0 mM, all from Sigma. All the treatments were performed for 24 h at 37 °C.

### 2.2. Transfections

For experiments, 15,000 cells/well were seeded on 8-well Ibidi µ-slides (IbiTreat, Ibidi, Martinsried, Germany). The transient transfections were performed with TurboFect (Thermo Fisher Scientific) according to the manufacturer’s instructions. The medium was replaced with fresh complete medium 16 h later. The construct expressing a GFP-HAS3 fusion protein used for transient transfections was previously described (see [23]).

### 2.3. Actin Staining

After transfections or inductions (24 h), the cells were washed with 0.1 M sodium phosphate buffer, pH 7.4 (PB), fixed with 4% paraformaldehyde in PB for 20 min and washed again. The fixed cells were permeabilized for 15 min with 0.1% Triton X-100 with 1% BSA, blocked with 1% BSA for 20 min at room temperature, and then incubated for 20 min with Phalloidin-iFluor 594 Reagent (Abcam, Cambridge, UK), washed with PB, and stored at 4 °C.

### 2.4. Confocal Microscopy

The fluorescent images were obtained with a Zeiss Axio Observer inverted microscope (63 × NA 1.4 oil objective) equipped with a Zeiss LSM 800 confocal module (Carl Zeiss Microimaging GmbH, Jena, Germany). Image processing, including three-dimensional rendering, was performed using ZEN software (Carl Zeiss Microimaging GmbH). The GFP was excited with the 488 nm laser and phalloidin 594 with the 555 nm laser. In addition, suitable excitation and emission filters were used for both fluorophores.

### 2.5. FiloQuant Analysis

For analysis of filopodia, stacks of 6–10 optical sections at 290 nm intervals from the bottom area of the cells were captured. To analyze filopodia density and length, FiloQuant plugin for the ImageJ software (1.52i) was utilized [20]. The manual tracking and single image tool were utilized to analyze the filopodia from maximum intensity projections created from the stacks of optical sections. Single image FiloQuant was used to detect and measure the length and number of filopodia. Filopodia density was defined as a ratio of the number of detected filopodia to cell edge length from the FiloQuant analysis.

### 2.6. Hyaluronan Assay

Subconfluent cell cultures on 24-well plates were used to measure the cellular hyaluronan secretion levels. After treatments or transient transfections, fresh medium was changed, and cells were cultured for 24 h before the cells were counted and the media harvested for the sandwich type assay as described previously [15]. (Rilla et al. 2008).

### 2.7. Statistical Analyses

Statistical analyses were carried out using the GraphPad Prism version 5.00 for Windows (Graph-Pad Software, San Diego, CA, USA). The significance of differences between groups was tested using one-way analysis of variance (ANOVA) with Dunnett’s post hoc tests or using Student’s *t*-test. Differences were considered significant when *p* < 0.05.

## 3. Results

### 3.1. GFP-HAS3-induced Filopodia are Sensitive to Fixation

**Figure 1:**
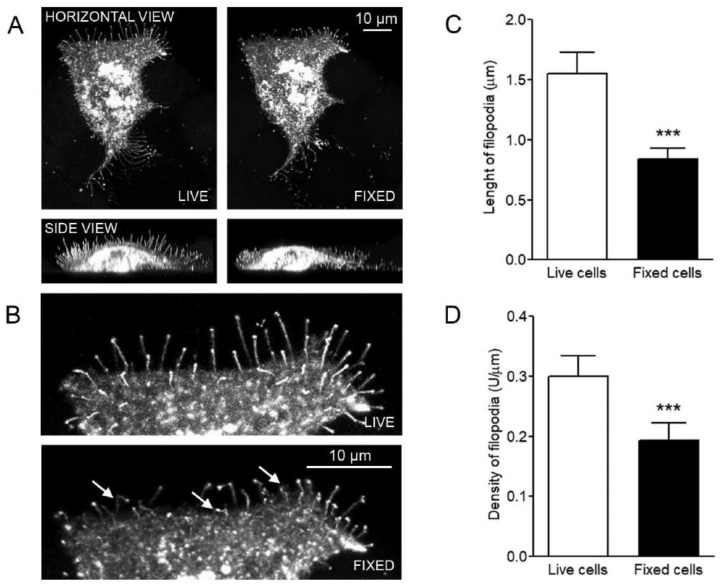
Numbers and length of filopodia are decreased after fixation. (**A**) The same GFP-hyaluronan synthase 3 (HAS3) expressing cell before (live) and after fixation with 4% paraformaldehyde (fixed). (**B**) A higher magnification from one edge of the cell. FiloQuant measurement of (**C**) filopodia length and (**D**) relative density in live and fixed cells (*n* = 25 cells for both groups). *** *p* < 0.001. Unpaired *t*-test. Magnification bars = 10 µm.

First, we analyzed MCF-7 breast cancer cells with inducible GFP-HAS3 expression to assess the effect of fixation on the length and density of filopodia. Figure 1A shows a maximum intensity projection created from stacks of confocal optical sections of the same GFP-HAS3 expressing cell before and after fixation. As shown in previous reports [12,13], GFP-HAS3 was strongly enriched in filopodial tips both in live and fixed cells. After fixation, most filopodia were notably shortened or collapsed (arrows in Figure 1B). The side views demonstrate that dorsal filopodia were especially collapsed upon fixation. The quantitative analysis shows that there is a significant decrease in both the length (Figure 1C) and density (Figure 1D) of filopodia after fixation of the cells.

### 3.2. Comparison of EGFP-HAS3 and Phalloidin Signal in the Detection of Filopodia with FiloQuant Software

**Figure 2:**
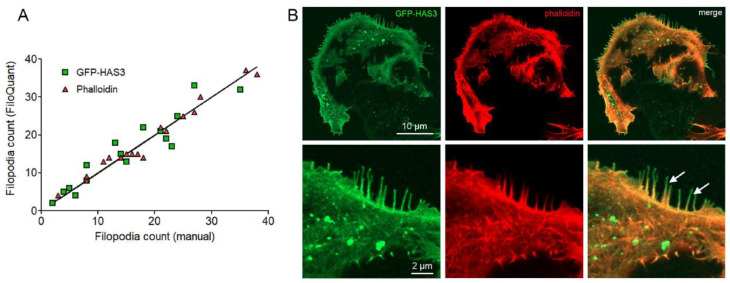
Precision in filopodia detection with EGFP-HAS3 and phalloidin signal in fixed cells. (**A**) The amount of filopodia detected with FiloQuant analysis is plotted against the manually annotated filopodia number for each cell edge by using EGFP-HAS3 signal (green squares, R^2^ = 0.8964) and phalloidin signal (red inverted triangles, R^2^ = 0.9717). (**B**) Images illustrating EGFP-HAS3 signal and phalloidin signal; arrows in (B) point filopodia in the edge of MCF-7 cell expressing GFP-HAS3 (*n* = 16 cells for both groups).

Next, the feasibility of the GFP-HAS3 signal was compared to the phalloidin signal in FiloQuant analysis of inducible MCF-7 cells. We compared the GFP-HAS3 signal with actin staining (phalloidin) in the same cells, by performing both manual tracking with a single image tool of FiloQuant and manual counting of the number of filopodia by utilizing both signals (Figure 2A). The results showed that the phalloidin is more reliable for FiloQuant analysis, as linear regression showed that the manual counting gave values closer to those received with the FiloQuant analysis (R^2^ = 0.9717) as compared to the GFP signal (R^2^ = 0.8964). However, the slopes were not statistically different (*p* = 0.8321). Visualization of both signals indicate that the overall length of filopodia is higher with GFP-HAS3 signal than with actin signal (arrows in Figure 2B). This difference is because plasma membrane-localized GFP-HAS3 signal accumulates into the tips of filopodia, while cytoplasmic actin signal detected by phalloidin weakens towards the tips.

### 3.3. Quantification of the Effect of HAS3 Expression on the Density and Average Length of Filopodia

**Figure 3:**
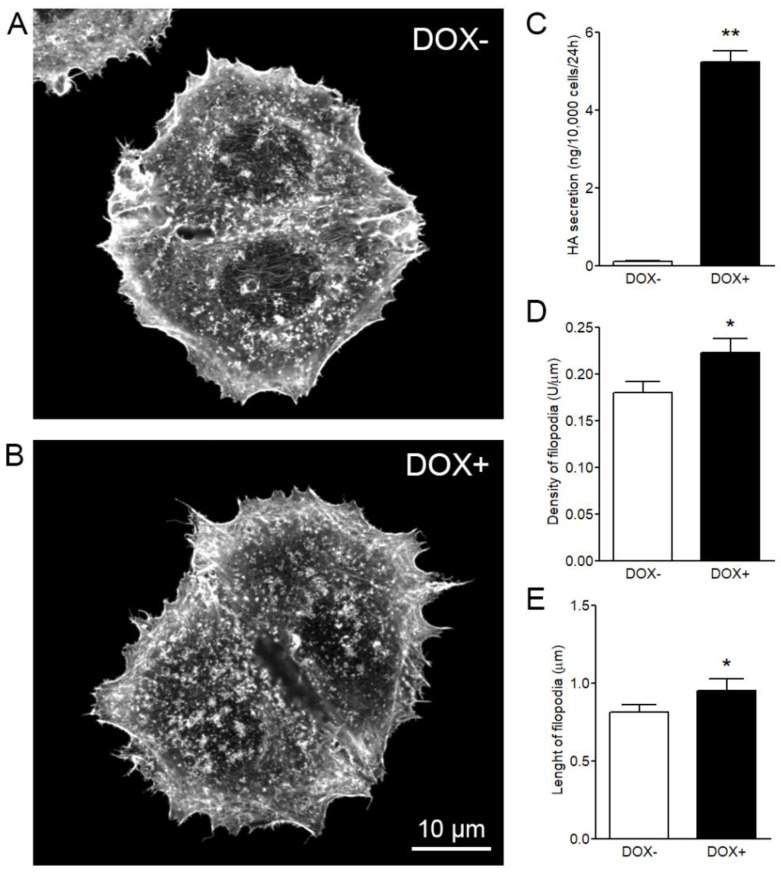
Analysis of stable MCF-7 cells with inducible expression of GFP-HAS3 in fixed and phalloidin-stained cells. Examples of analyzed cells (**A**) without and (**B**) with induction of GFP-HAS3 expression by doxycycline. (**C**) The levels of hyaluronan synthesis, (**D**) the average density, and (**E**) the average length of filopodia without and with induction. Student’s *t*-test, * *p* < 0.05. ** *p* < 0.01 (*n* = 5 independent experiments with 16–28 cells/group in each experiment).

Based on the observations described above, most of the further measurements were performed by using phalloidin staining. HAS3 overexpression has shown to increase the length of filopodia in many different cell lines [12,24,25], but no quantitative data have been previously published. Analysis of stable MCF-7 cells with inducible expression of GFP-HAS3 was performed in fixed and phalloidin stained cells (Figure 3A,B). The data showed that induced hyaluronan synthesis by HAS3 (Figure 3C) significantly increased both density (Figure 3D) and average length (Figure 3E) of filopodia.

### 3.4. Both Length and Density of HAS3-Induced Filopodia are Dependent on Glucose Levels

**Figure 4:**
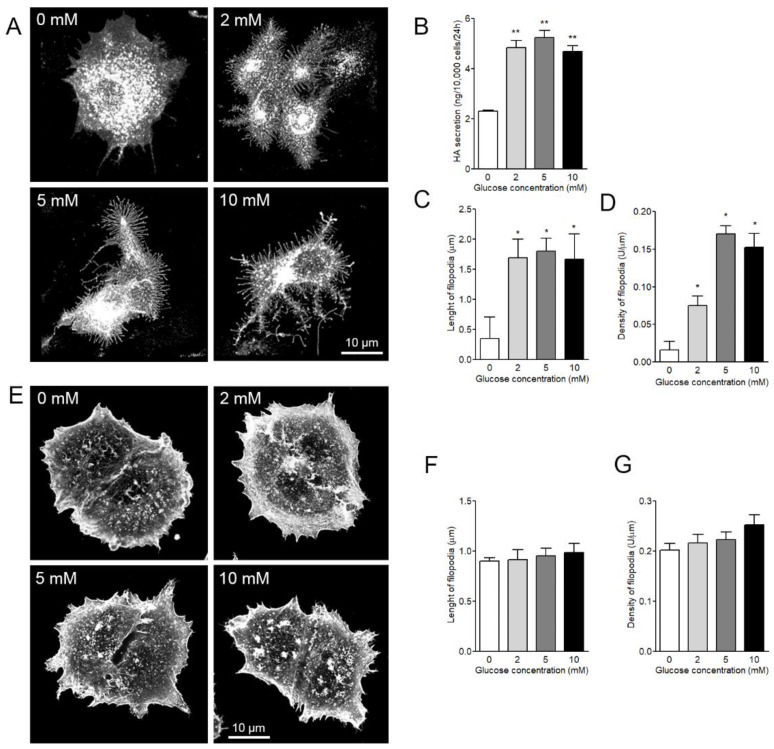
Both length and density of HAS3-induced filopodia are dependent on glucose levels. (**A**) Examples of live MCF-7 cells grown in different glucose concentrations. (**B**) The levels of hyaluronan secretion in different glucose concentrations and (**C**) FiloQuant analysis of the length and (**D**) density of filopodia in live cells. (**E**) Examples of fixed and phalloidin-stained MCF-7 cells grown in different glucose concentrations. FiloQuant analysis on the (**F**) length and (**G**) density of fixed and phalloidin-stained cells grown in the same concentrations of glucose. One-way ANOVA, * *p* < 0.05, ** *p* < 0.01, as compared to control. (*n* = 5 independent experiments with 18–30 cells/group in each experiment).

Since the levels of glucose are known to regulate hyaluronan synthesis rate [24,26], the effect of glucose concentration in the culture media of GFP-HAS3 overexpressing MCF-7 cells on filopodial growth was quantified. Live MCF-7 cells induced to express GFP-HAS3 and grown in different glucose concentrations (Figure 4A) showed clear and significant dependence of hyaluronan secretion levels on glucose supply (Figure 4B). In addition, filopodial length (Figure 4C) and density (Figure 4D) were significantly dependent on glucose supply. To confirm these findings and eliminate bias introduced by possible changes in the localization of GFP-HAS3 signal when cultured in different glucose concentrations, the same analysis was performed after fixation and phalloidin staining of the cells (Figure 4E). The same trend was detected in fixed, phalloidin-stained cells on both filopodia length (Figure 4F) and density (Figure 4G), but as expected, the differences were less prominent as compared to live cells. The different levels in average density and length of filopodia in live and fixed cells are due to fixation and different labels used in measurements.

### 3.5. Effect of Glucosamine, Mannose, and 4-MU Treatment on HAS3-Induced Filopodia Formation

**Figure 5:**
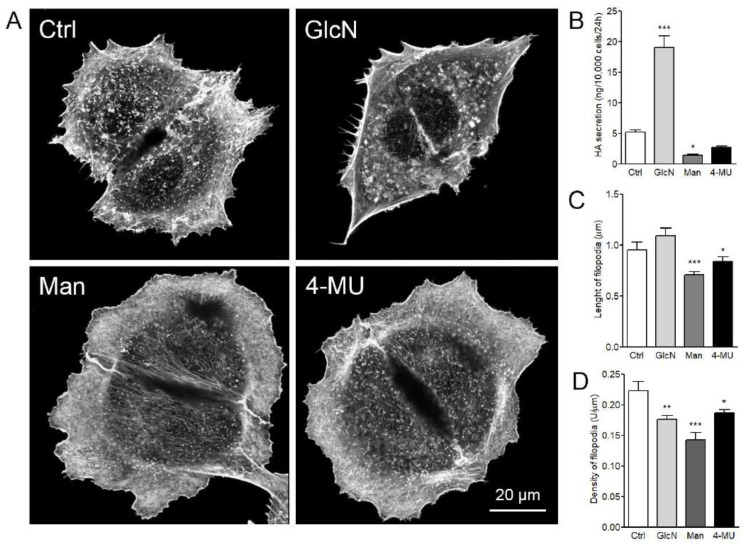
Effect of glucosamine, mannose, and 4-methylumbelliferone (4-MU) treatments on the formation of HAS3-induced filopodia. (**A**) Examples of analyzed GFP-HAS3 expressing fixed and phalloidin-stained MCF-7 cells with different treatments are shown in and (**B**) analysis of hyaluronan secretion, (**C**) length, and (**D**) density of filopodia. One-way ANOVA, * *p* < 0.05, ** *p* < 0.01, *** *p* < 0.001, as compared to control (*n* = 5 independent experiments with 16–28 cells/group in each experiment).

Next, the hyaluronan secretion levels in GFP-HAS3 expressing MCF-7 cells were manipulated by factors that are known to regulate the levels of sugar precursors for hyaluronan synthesis by different mechanisms (Figure 5A). Hyaluronan secretion levels were significantly increased by glucosamine (2 mM) treatment and decreased when treated by mannose (2 mM) or 4-MU (1 mM) (Figure 5B). Both mannose and 4-MU had a clear effect on cellular morphology, resulting in a flattened shape as compared to the control (Figure 5A). There was a small increase in the average length of filopodia upon addition of glucosamine (Figure 5C), but a significant decrease was detected in the filopodial density (Figure 5D). Mannose significantly decreased hyaluronan secretion (Figure 5B), average length (Figure 5C), and density (Figure 5D) of filopodia. 4-MU inhibited hyaluronan secretion, but it was not statistically significant (Figure 5B). However, 4-MU significantly decreased both the average length (Figure 5C) and density (Figure 5D) of filopodia.

### 3.6. Effect of CD44 Expression on Filopodia Length and Numbers in MKN74 Cells

**Figure 6:**
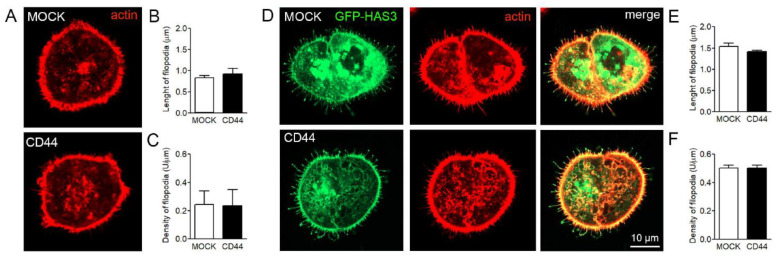
Effect of CD44 expression on filopodia length and density in MKN74 cells. (**A**) Examples of fixed and phalloidin-stained MKN74-MOCK and MKN74-CD44 cells and (**B**) average length and (**C**) density of filopodia. The effect of GFP-HAS3 expression on the filopodia of MKN74 cell lines was studied by transient transfections, followed by fixation and phalloidin staining. (**D**) Examples of analyzed cells and the (**E**) average length and (**F**) density of filopodia are shown (*n* = 3 independent experiments with 14–41 cells/group in each experiment).

To study the effect of hyaluronan receptor CD44 on filopodial growth, two versions of human gastric cancer cell line MKN74, MOCK (CD44-negative) and CD44 (CD44 overexpression), were utilized (Figure 6A). The characterization of hyaluronan metabolism of MKN74 has shown that CD44 overexpression induces binding of hyaluronan to the plasma membrane, especially around filopodia [22]. However, the level of secreted hyaluronan was slightly higher in MOCK cells, the average amount being 2.5 ng/10,000 cells, while CD44+ cells produced 1.9 ng/10,000 cells of hyaluronan during 24 h [22]. The FiloQuant analysis showed no significant difference in length (Figure 6B) or density (Figure 6C) of filopodia between the two cell lines. Further, to find out whether CD44 is required for the formation of HAS3-induced filopodia, both MOCK and CD44+ MKN74 cell lines were transiently transfected with GFP-HAS3 construct (Figure 6D). Both cell lines were able to form filopodia with high accumulation of GFP-HAS3 in filopodia and their tips (Figure 6D). Analysis of phalloidin staining showed that HAS3 expression induced the average length (Figure 6B,E) and density (Figure 6C,F) of filopodia in both cell lines, but no difference either in average length (Figure 6E) or density (Figure 6F) of filopodia between CD44 negative and CD44 expressing cells was detected.

### 3.7. HA Secretion and Filopodial Growth in MCF10A and MCF10CA Breast Cancer Cell Lines

**Figure 7:**
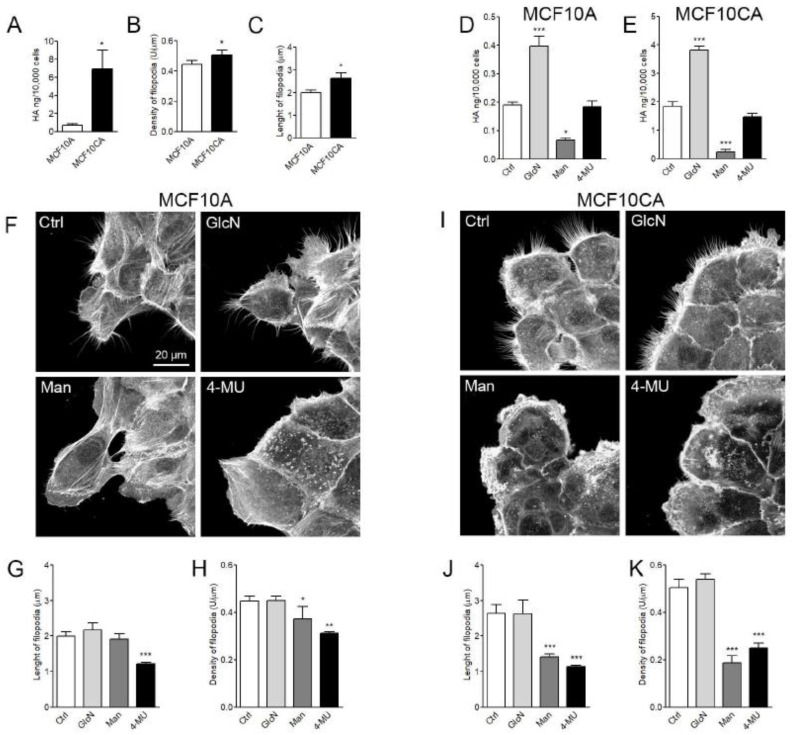
Hyaluronan (HA) secretion and filopodial growth in MCF10A and MCF10CA breast cancer cell lines. (**A**) HA secretion levels, (**B**) density, and (**C**) length of filopodia of fixed and phalloidin-stained MCF10 cell lines. Effect of different treatments on hyaluronan secretion of (**D**) MCF10A and (**E**) MCF10CA cells. (**F**) Examples of MCF10A cell cultures and (**I**) MCF10CA cell cultures after different treatments. (**G**) Average length and (**H**) density of filopodia in MCF10A cells, and (**J**) length and (**K**) density of filopodia in MCF10CA cells after different treatments. Ctrl = control, GlcN = glucosamine (2 mM), Man = mannose (20 mM), and 4-MU = 4-methylumbelliferone (1 mM). Student’s *t*-test was utilized to compare the cell lines (A–C) and one-way ANOVA compared the treatments (D, E, G, H, J and K). * *p* < 0.05, ** *p* < 0.01, *** *p* < 0.001 (*n* = 5 independent experiments with 18–43 cells/group in each experiment).

Two human cell lines, immortalized breast epithelial cells MCF10A and tumorigenic MCF10CA cells, were utilized to compare hyaluronan secretion levels and growth of filopodia in normal and malignant cells. The malignant MCF10CA cells produced significantly higher levels of hyaluronan than MCF10A cells (Figure 7A). As expected, the average density (Figure 7B) and length (Figure 7C) of filopodia were significantly higher in MCF10CA cells as compared to MCF10A cells. The glucosamine treatment enhanced hyaluronan synthesis levels in both MCF10A (Figure 7D) and MCF10CA (Figure 7E) cells. Mannose significantly inhibited hyaluronan synthesis in both cell lines, but 4-MU did not have a significant effect (Figure 7D,E). Glucosamine did not have a significant effect on the growth of filopodia in either of the cell lines (Figure 7G–K). The inhibitory effect of mannose and 4-MU in average density of filopodia was more pronounced in MCF10CA cells (64% and 50%, respectively) than in MCF10A cells (16% and 20%, respectively) (Figure 7G,J). The same trend was found in the average length of filopodia: 4-MU inhibited the average length of filopodia by 40% in MCF10A and 57% in MCF10CA, while mannose inhibited it 4% in MCF10A and 47% in MCF10CA cells (Figure 7H,K).

## 4. Discussion

### 4.1. FiloQuant Analysis of GFP-HAS3-Induced Filopodia

FiloQuant is a novel, specific software for quantification of the amount and length of filopodia [20]. We started this study by evaluating the suitability of FiloQuant software for quantitative analysis of HAS-induced filopodia. As reported before, the HA-rich pericellular zones are highly sensitive for fixation [27]. Therefore, HA-dependent filopodia are also fragile structures and very sensitive for sample processing, such as fixation and dehydration [12]. However, the pitfalls of the analysis of HA-induced filopodia in live cells are the movements of filopodia during imaging, and possible phototoxicity, which may interfere with the analysis. The results of this study provide quantitative evidence that cell fixation can promote the shrinkage or collapsing of filopodia, yielding lower average length and density detections when compared to those observed in live conditions. Therefore, the effect of HAS3 overexpression on filopodia growth is more clearly detectable in live cells than in fixed cells. Additionally, FiloQuant is optimal for quantification of lateral filopodia attached to the bottom of the plate, and not suitable for dorsal filopodia, which are a typical feature of HAS3-expressing cells [12]. However, the number and length of lateral filopodia represent the relative number and length of all filopodia in each cell. Additionally, cytoplasmic actin staining with phalloidin underestimates the length of filopodia as compared to GFP-HAS3 signal localized to the plasma membrane and especially to the tips of filopodia. However, the analyses with fixed, phalloidin-stained cells show the relative differences between analyzed groups and are thus suitable for reliable comparative analysis. In summary, the results demonstrate that filopodia are notably affected by sample processing, such as fixation.

### 4.2. Filopodial Growth Is Associated with Hyaluronan Synthesis Activity

The results of this study indicate, for the first time by quantitative analysis, the association of hyaluronan synthesis activity with the growth of filopodia. Typically, cancer cells have more filopodia than normal cells, promoting migration, adhesion, invasion, and metastasis [28]. Another typical hallmark of cancer cells is that they secrete hyaluronan more actively than normal cells [29]. Many previous studies performed by our group and others suggest that these cellular features are connected to each other. Cell types that have especially long and numerous plasma membrane protrusions, such as neuroblastoma cells [30], smooth muscle cells [15], human fibroblasts [31,32], and fibroblasts of Shar Pei dogs with high HAS2 expression [33] have typically high hyaluronan secretion capacity. Even more direct evidence on the effect of hyaluronan synthesis on the growth of filopodia is the rapid regrowth of filopodia after blocking and releasing of hyaluronan synthesis [12,13]. In addition to filopodia function as extracellular matrix sensors and remodeling, their formation is also influenced by the surrounding extracellular matrix [34]. As an abundant, big, multifunctional, and water binding molecule that forms the structural basis of the pericellular and intercellular matrix, hyaluronan is one of the most important extracellular modifiers of the plasma membrane shape.

It is now clear that hyaluronan synthesis is associated with filopodial growth. The numeric data of this work on hyaluronan secretion levels and filopodia density and length are summarized in Table 1. Nevertheless, the exact mechanism whereby HA synthesis induces such a prominent and dynamic change in plasma membrane morphology is still substantially unresolved. The most convincing theory comes from a biophysical point of view. It is known that high levels of HA on the plasma membrane increase hydrostatic pressure [35] and provide energy for regulation of the plasma membrane curvature [36]. This energy facilitates filopodia formation [13] by creating a pulling force with which to modulate the intracellular actin-myosin-based contractile cytoskeletal machinery [37]. It has also been suggested that the packing of HA chains in cylindrical and spherical brushes on protrusions leads to higher energy gains than can be achieved with the planar brushes associated with plain plasma membranes [36]. On the other hand, the highly hydrated hyaluronan layer outside of the plasma membrane gives mechanical support to maintain filopodia, especially those not attached to the substratum [13]. This is supported by the finding that hyaluronidase digestion results in identical morphological change on filopodia as a fixation [13], suggesting that the dehydration effect results in the collapse of the filopodia in both cases. The physical properties of hyaluronan layer are affected by molecular weight of hyaluronan chains [36], but no data are available on the effect of the size of hyaluronan on its ability to form and maintain filopodia.

### 4.3. Involvement of CD44 in Filopodia Formation

Overexpression of CD44 has been observed in many types of cancer, and the interaction between CD44 and ezrin, radixin, and moesin links the actin cytoskeleton to the plasma membrane and the extracellular matrix [38]. These interactions are critical for CD44 function in cell–cell adhesion and cell motility related to inflammatory cell functions as well as in tumor growth and metastasis. However, as a protein that increases cell motility and invasion of cancer cells, the role of cell surface adhesion protein CD44 in the formation of filopodia has been minimally studied. To study the effect of CD44 on filopodial growth, human gastric cancer cell line MKN74 was utilized [22]. The results of this study showed that CD44 expression had no effect on the formation of filopodia of MKN74 cells. No significant differences either in average length of filopodia or density were detected in CD44-positive in comparison to CD44-negative cell lines. Both cell lines were also capable to form HAS3-induced filopodia. This result supports earlier findings suggesting that CD44 is not required for formation of filopodia [12,14,22]. Although filopodia have been shown to be strongly CD44-positive, the disruption of the hyaluronan–CD44 interactions [12] or inhibition of CD44 had no effect [14] on the number or length of filopodia. The role of another hyaluronan receptor, RHAMM, on filopodial growth seems to be contradictory; there is evidence on its impact on filopodial growth of esophageal squamous cell carcinoma [14], but not breast adenocarcinoma [12] cells.

However, previous findings suggest that overexpression of CD44 increases the number and length of filopodia in cells of the nervous system [30,39]. In neuroblastoma cells CD44 overexpression induced longer filopodia-like structures, which was associated with increased cell invasion [30]. In a study with neural precursor cells, overexpression of CD44 increased the number and length of filopodia, with enhanced invasiveness [39]. Interestingly, CD44 regulates functional and structural plasticity of dendritic spines, specific protrusions that develop from dendritic filopodia [40]. Furthermore, in a recent report, CD44 was reported to be crucial for microtentacles, specific protrusions formed by glioblastoma cells in hyaluronan-rich extracellular matrix (ECM) [41]. Our results confirm that CD44 is not essential for the formation of filopodia in MKN74 cells. However, previous findings described above suggest that it may be a significant factor in the regulation of filopodia and other protrusions in other cell types.

## 5. Conclusions

Filopodia are thin and fragile structures that are challenging to analyze but have multiple functions in normal and tumor cells. One of the most intriguing factors suggested to regulate the growth of filopodia is hyaluronan synthesis activity. This work shows for the first time quantitative data on the correlation of filopodia and hyaluronan metabolism in cancer cells. This study also summarizes the reciprocal association between hyaluronan synthesis activity and growth of filopodia upon different treatments. We show quantitative analysis of filopodia in three human cancer cell lines with induced expression of HAS3, CD44, and variable hyaluronan synthase activity by manipulating the levels of sugar precursors for hyaluronan building blocks. The results of this study strengthen the hypothesis that in addition to actin cytoskeleton, which is crucial for filopodial structure and dynamics, external factors of the extracellular niche, such as hyaluronan, also have a central role in their formation. In the future, it will be important to utilize three-dimensional culture conditions to understand how nonfibrillar HA-rich matrices lacking integrin ligands support the formation of filopodia.

## Figures and Tables

**Table 1:** Hyaluronan secretion levels and average length and density of filopodia in different cell lines producing various levels of hyaluronan endogenously, or via different manipulations.

Cell Line	Hyaluronan Secretion (ng/10,000 cells/24 h)	Average Density of Filopodia (U/µm)	Average Length of Filopodia (µm)
MCF-7-HAS3 Dox-	0.11	0.18	0.82
MCF-7-HAS3 0 mM Glc	2.30	0.20	0.90
MCF-7-HAS3 2 mM Glc	4.84	0.21	0.91
MCF-7-HAS3 5 mM Glc	5.23	0.22	0.95
MCF-7-HAS3 10 mM Glc	4.69	0.25	0.98
MCF-7-HAS3 + GlcN	19.0	0.18	1.10
MCF-7-HAS3 + Man	1.41	0.14	0.70
MCF-7-HAS3 + 4-MU	2.70	0.19	0.84
MKN74 MOCK	2.50	0.25	0.80
MKN74 CD44	1.90	0.24	0.90
MCF10A	0.19	0.45	1.99
MCF10A + GlcN	0.40	0.45	2.16
MCF10A + Man	0.07	0.37	1.92
MCF10A + 4-MU	0.18	0.31	1.22
MCF10CA	1.84	0.50	2.64
MCF10CA + GlcN	3.81	0.54	2.62
MCF10CA + Man	0.24	0.19	1.40
MCF10CA + 4-MU	1.46	0.25	1.14
